# Multivendor Spectral-Domain Optical Coherence Tomography Dataset, Observer Annotation Performance Evaluation, and Standardized Evaluation Framework for Intraretinal Cystoid Fluid Segmentation

**DOI:** 10.1155/2016/3898750

**Published:** 2016-08-04

**Authors:** Jing Wu, Ana-Maria Philip, Dominika Podkowinski, Bianca S. Gerendas, Georg Langs, Christian Simader, Sebastian M. Waldstein, Ursula M. Schmidt-Erfurth

**Affiliations:** ^1^Christian Doppler Laboratory for Ophthalmic Image Analysis, Department of Ophthalmology and Optometry, Medical University of Vienna, Vienna, Austria; ^2^Christian Doppler Laboratory for Ophthalmic Image Analysis, Computational Imaging Research Lab, Department of Biomedical Imaging and Image-Guided Therapy, Medical University of Vienna, Vienna, Austria

## Abstract

Development of image analysis and machine learning methods for segmentation of clinically significant pathology in retinal spectral-domain optical coherence tomography (SD-OCT), used in disease detection and prediction, is limited due to the availability of expertly annotated reference data. Retinal segmentation methods use datasets that either are not publicly available, come from only one device, or use different evaluation methodologies making them difficult to compare. Thus we present and evaluate a multiple expert annotated reference dataset for the problem of intraretinal cystoid fluid (IRF) segmentation, a key indicator in exudative macular disease. In addition, a standardized framework for segmentation accuracy evaluation, applicable to other pathological structures, is presented. Integral to this work is the dataset used which must be fit for purpose for IRF segmentation algorithm training and testing. We describe here a multivendor dataset comprised of 30 scans. Each OCT scan for system training has been annotated by multiple graders using a proprietary system. Evaluation of the intergrader annotations shows a good correlation, thus making the reproducibly annotated scans suitable for the training and validation of image processing and machine learning based segmentation methods. The dataset will be made publicly available in the form of a segmentation Grand Challenge.

## 1. Introduction

Spectral-domain optical coherence tomography (SD-OCT) is the most important ancillary test for the diagnosis of sight degrading diseases such as retinal vein occlusion (RVO), age-related macular degeneration (AMD), and glaucoma [1]. SD-OCT is a noninvasive modality for acquiring high resolution, 3D cross-sectional volumetric images of the retina and the subretinal layers, in addition to retinal pathology such as intraretinal fluid, subretinal fluid, and pigment epithelial detachment [2, 3]. Detection and segmentation of such pathologies are an important step in the diagnosis of disease severity and treatment success, as well as an early stage towards the accurate prediction of both [4, 5]. The detection of intraretinal cystoid fluid (IRF) is a particularly important indicator of disease severity and change in exudative macular disease as increased retinal thickness has shown to correlate with poor visual acuity [6]; thus automated detection and segmentation methods are required to employ “big data” in visual acuity and treatment progression prediction. Thus IRFs have been chosen as the basis for this multivendor reference dataset and grader performance assessment [7, 8]. 

At the time of writing, there is no publically available dataset of SD-OCT scans acquired from multiple SD-OCT devices and featuring a wide variety of IRF appearances, with an accompanying expertly annotated ground truth, that is to say, manually annotated IRF regions by trained individuals. Such a dataset is important for the development of novel segmentation algorithms as it allows for the training and testing of new systems with a general reference. In the current literature, methods of IRF segmentation are limited, using training and validation datasets that are not always publically available [9–12]. This results in difficulty in reproducing results for comparative purposes, which in addition do not always use the same evaluation measures. Equally important is the reproducibility of IRF annotations used to construct the reference standard. High interobserver agreement is necessary; however this is difficult due to the challenging nature of manual IRF delineation. The combination of a reproducibly annotated dataset and evaluation framework will facilitate the consistent and uniform comparison of newly developed and current methods through standardized measures of segmentation accuracy [13]. Furthermore, this would allow methods to be assessed as part of a segmentation challenge [14, 15], an important and effective means by which novel methods are developed in not only medical imaging research but also many other fields in computer vision. This may facilitate a better understanding of the positive and negative aspects of each developed method in an effort to improve performance, as well as opening avenues for further development or collaboration.

Thus the purpose of this work is to create a multivendor SD-OCT dataset comprised of clinically representative scans with IRFs annotated by multiple expert graders. This work will show the reproducibility of the annotations, suitable for use as a reference standard to both train and validate IRF segmentation methods. Furthermore, a standardized evaluation framework for IRF segmentation is presented.

## 2. Materials and Methods

### 2.1. Dataset

The dataset constructed here is comprised of 30 distinct SD-OCT scans from four major OCT devices used in ophthalmology (Zeiss Cirrus, Heidelberg Spectralis, Topcon 3D 2000, and Nidek RS3000) in the proportions described in Table 1. The image datasets were selected from the image database of Vienna Reading Center (VRC), featuring large datasets from several international phase II and III pharmaceutical trials in retinal disease. The individual images were chosen by medical experts in order to reflect a representative distribution of OCT scanners, acquisition settings, disease stages, and image quality.

This study was conducted in compliance with the tenets set forth in the declaration of Helsinki. The trials from which the scans were taken were approved by the institutional review board of the Medical University of Vienna. All patients gave written consent for participation in the respective trial and all data was appropriately anonymized.

The dataset is further divided into 15 training scans and 15 testing scans chosen to be representative of the wide variety of scans seen in the clinical environment, in addition to the wide variety of IRF appearances and distributions. Both the training and testing subsets comprised 4 scans per vendor aside from Nidek with 3. Each scan within this dataset has been explicitly chosen to contain a wide variety of IRF sizes, shapes, and appearances. This is particularly important for algorithm training (such as that of machine learning techniques) as methods will need to learn the variety of possible cyst appearances across different devices while factoring in the noise pattern and signal response variation across different devices. All 15 training scans have been annotated on each individual slice comprising the OCT volume (henceforth known as a B-scan) by two distinct expert graders at the Christian Doppler Laboratory for Ophthalmic Image Analysis (OPTIMA), Medical University of Vienna, who have been trained to identify IRFs using a criteria explained in the following section.

**Figure 1:**
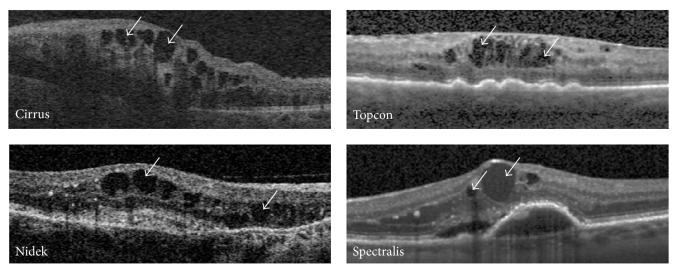
Exemplar retinal B-scans from 4 SD-OCT devices showing variations in noise and appearance. White arrows indicate exemplar IRFs.

The testing set is intended for validation of IRF segmentation systems and thus also contains the same spectrum of IRF appearances, sizes, and shapes as seen in the training subset, in addition to normal cases to act as control images.  Figure 1 presents exemplar B-scans from each of the 4 devices, exemplifying the varying signal and noise and IRF appearance variations (indicated by the white arrows).

**Figure 2:**
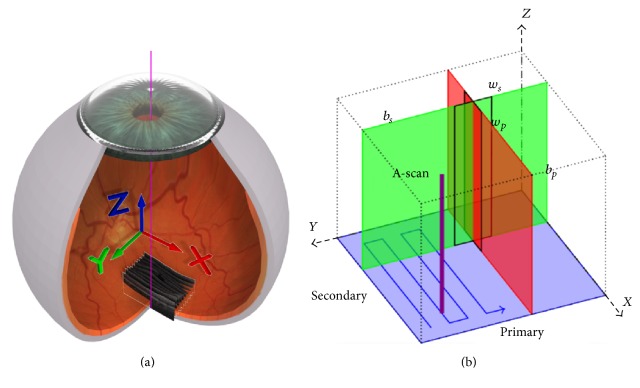
(a) Retinal OCT scan coordinate space in relation to anatomical eye. (b) OCT scan pattern representing the red, green, and blue colored planes shown in (a) [16].

Each retinal OCT volume is approximately 6 × 6 × 2 mm^3^ and centered on the macula. The coordinate system used to represent the retinal volume is shown in Figure 2 [16].  Figure 2(a) demonstrates the location of the B-scans in relation to the anatomical eye and the respective *X*, *Y*, and *Z* image planes in red, green, and blue. In Figure 2(b) the primary (*b*_*p*_) and secondary (*b*_*s*_) scan directions are depicted, in addition to their relationships with the major image planes. Using the same color coding system as described previously, the red B-scan can be seen, in addition to the perpendicular green A-scan. The windows defined by *w*_*p*_ and *w*_*s*_ are not utilized here. Furthermore, Figure 2(a) shows the raster scan pattern (blue arrow) utilized by the OCT devices used to acquire the scans for this dataset. Dependent on device, the physical dimensions equate to 200 × 200 × 1024, 256 × 256 × 885, 512 × 128 × 885, 512 × 128 × 1024, or 512 × 49 × 496 pixels.

### 2.2. IRF Annotation

**Figure 3:**
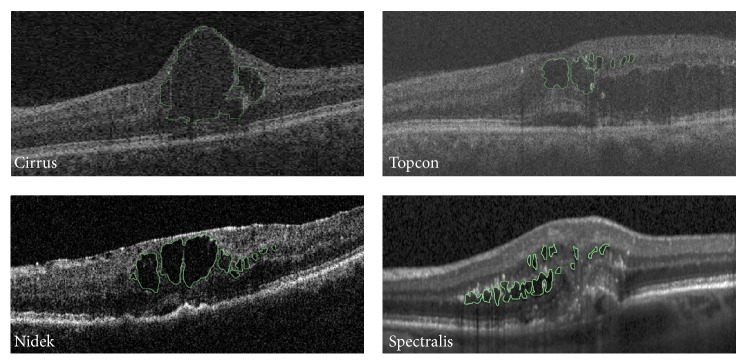
Exemplar annotated B-scans showing annotated cysts in green.

Annotation was performed using a proprietary system developed at the OPTIMA Lab with functionality to perform manual pixel level annotations of retinal SD-OCT scans. Annotation is performed in the B-scan plane, examples of which are shown in Figure 3 for each device where the annotated IRF outline is shown in green. Not only do the examples in Figure 3 exemplify the varying size and appearance of IRFs in SD-OCT, but also it can be seen that scans from different vendors vary, sometimes greatly, with respect to image quality, signal-to-noise ratio (SNR), and contrast, thus making the task of manual IRF segmentation very difficult and time-consuming.

Each grader was tasked with manually delineating the IRF structures that were visible to them in each B-scan of a volume using free-hand drawing with a stylus and tablet.

The criteria the graders used to analyze IRFs were as follows:

- *Shape/intensity:* the IRF shape spectrum is broad, ranging from circular/oval to an amorphous blob. However, IRF intensity is generally low due to the attenuation of light as the medium is primarily liquid.
- *Distinction:* IRFs usually have distinct borders separating their interior with the surrounding tissue. However, this is dependent on the scan image quality and the presence of noise.
- *Continuity:* IRFs are three-dimensional objects and as such may be present across multiple contiguous B-scans. However, this is dependent on IRF size and the B-scan slice thickness used at acquisition by the device and study protocol.
- *Position:* IRFs which significantly affect visual acuity are generally located in and around the fovea of macula centered retinal OCT scans, which is the functional center of vision.

This annotation process stores the manually delineated regions on each B-scan, *S*_Bscan_(*Z*, *X*), within a separate volume containing the positions of the annotated cysts, *V*(*Z*, *X*, *Y*), extractable using various computational means. For usage purposes, the annotated IRFs are extracted using MATLAB (The Mathworks Inc.) and stored using the standardized XML format [17] and the coordinate system described previously and in Figure 2.

Figure 3 shows exemplar annotated IRFs from each of the four devices, outlining in green the annotated IRF structure(s). As can be seen, IRFs range in size and appearance, as well as location. In addition, Figure 3 demonstrates the challenging nature of manual human expert annotation of such objects given that cysts may be extremely small in size, with difficult-to-delineate boundaries, requiring approximately 150 hrs in total to annotate the 30 scans. The time intensive nature of manual IRF segmentation further illustrates the requirement for accurate and reproducible automated methods for IRF segmentation, as it is not feasible nor possible for human graders to perform this task accurately for such long periods or for large datasets.

### 2.3. Standardized Evaluation Framework

IRF segmentation algorithm results must be evaluated in a standardized way so that results from different methods are comparable. In addition, as IRFs are delineated by their boundaries, a relevant measure of accuracy is required to gauge system performance. Thus we propose the use of three initial measures: firstly area overlap with reference IRF positions, secondly distance from reference IRF boundaries, and thirdly the intersection-over-union which is also widely used in evaluating image segmentation. The first measure examines the overlap between system segmented IRF area results and reference standard, based on the Sørensen-Dice index (DSC) [18]. The second measure is based on the Hausdorff distance [19] which examines the distance between the system segmented IRF regions and ground truth. The third measure examines the overlap between system and reference IRF areas by computing the intersection divided by the union. The set of IRF coordinate points of all segmented IRFs on a given B-scan is defined as *S*_Bscan_(*Z*, *X*) and the reference IRF points for a given B-scan are defined as *R*_Bscan_(*Z*, *X*) where *Z* is the position on the vertical axis of the B-scan, *X* is the position on the horizontal axis of the B-scan, and *Y* is the B-scan in the volume (Figure 2):

(1) $$\begin{array}{c} O_{Bscan}=\frac{2\left|S_{Bscan}\cap R_{Bscan}\right|}{\left|S_{Bscan}\right|+\left|R_{Bscan}\right|}, \end{array}$$

where *O*_Bscan_ is the overlap for a specific B-scan:

(2) HSBscan,RBscan=max⁡hSBscan,RBscan,hRBscan,SBscan,

where *H* is the Hausdorff distance between sets *S*_Bscan_ and *R*_Bscan_:

(3) $$\begin{array}{c} I_{Bscan}=\frac{area\left(S_{Bscan}\cap R_{Bscan}\right)}{area\left(S_{Bscan}\cup R_{Bscan}\right)}, \end{array}$$

where *I*_Bscan_ is the intersection-over-union overlap for a specific B-scan.

Thus we compute the overlap between the reference annotation and system segmentation for a given B-scan using (1) resulting in a value within {0 ⋯ 1} where being closer to 0 represents poor overlap and being closer to 1 a high overlap, taking the mean over all B-scans with cysts to give the overlap for the entire volume (*O*_Volume_). We use the Hausdorff distance between point sets *S*_Bscan_ and *R*_Bscan_ as described by (2) to compute the distance between the ground truth and segmented IRFs for a given B-scan resulting in a pixel value (*H*_Bscan_). We compute the mean distance over all B-scans with IRFs to give the overall distance for the volume (*H*_Volume_). The intersection-over-union overlap between reference and system segmentation for a given B-scan is computed using (3) resulting in a value within {0 ⋯ 1}, where being closer to 0 represents poor overlap and being closer to 1 a high overlap. Again the mean over all B-scans with cysts is computed to give the overlap for the entire volume (*I*_Volume_).

**Figure 4:**
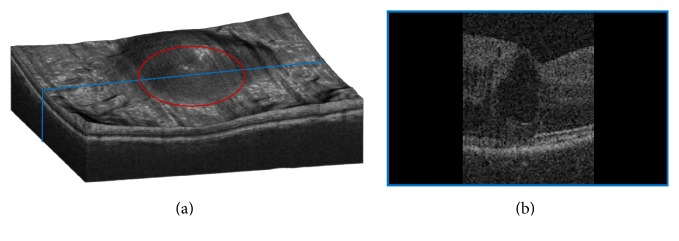
(a) Exemplar retinal OCT volume depicting the circular ROI in red. (b) Exemplar B-scan taken from the location represented in blue in (a).

In addition to the overall score resulting from the three quantitative measures mentioned here, system performance is further evaluated using two further criteria: clinical significance of the IRF and IRF size. Due to their composition and position, some IRFs may be more clinically significant to disease than others. These IRFs tend to be larger and located below and around the fovea, which is the functional center of vision [20]. Thus their size and position are used as classifiers with the central 3 mm circular region used as a mask *m* applied to the enface OCT image [21]. This is demonstrated in Figure 4 where the red circle in Figure 4(a) denotes the masked region displayed on a volume render of the retinal OCT. The B-scan seen in Figure 4(b) demonstrates post masking, where the blue line in Figure 4(a) denotes where the B-scan is located. Thus *O*_Mask_ and *H*_Mask_, respectively, denote the DSC overlap and Hausdorff distance for the masked region.

However, larger IRFs are generally much more visible; thus smaller IRFs are harder to delineate due to poor SNR and poor boundary distinction. Thus the second additional measure assigns a label to small cysts such that their segmentation accuracy is evaluated separately. For the purposes of this evaluation framework, a small IRF is assigned a physical minimum size (*μ*m), computed from the minimum IRF size as annotated by expert graders at the OPTIMA Lab. Thus *OS*_Volume_ and *HS*_Volume_ denote the DSC and Hausdorff distance of small IRFs per volume, and *OS*_Mask_ and *HS*_Mask_ denote the DSC and Hausdorff distance for small IRFs within the masked region. Small IRF size is defined by the minimum IRF size for each vendor in Table 2. It should be noted that a separate minimum IRF size has been identified per device; this is due to the interdevice image acquisition differences.

In summary, the ten measures defined to evaluate segmentation performance are as follows:

(1) Overall overlap using DSC, *O*_Volume_.
(2) Mean Hausdorff distance between IRF boundaries, *H*_Volume_.
(3) Intersection-over-union, *I*_Volume_.
(4) Measures 1, 2, and 3 within the central 3 mm masked region (Figure 4(a)), *O*_Mask_  and  *H*_Mask_.
(5) Measures 1, 2, 3, and 4 for small IRFs, *OS*_Volume_, *HS*_Volume_, *IS*_Volume_, *OS*_Mask_ , and  *HS*_Mask_.

## 3. Results

**Figure 5:**
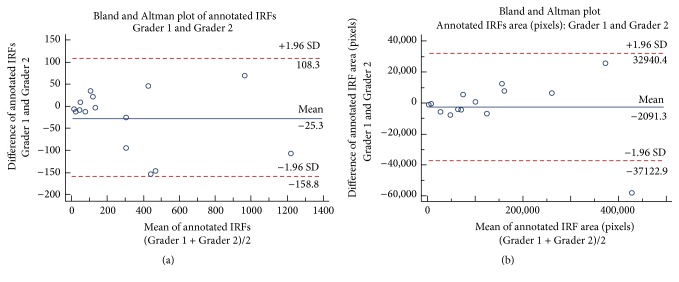
Bland Altman plots of annotated IRFs by the two graders. (a) Agreement of manual annotation between Graders 1 and 2 was good with a mean difference of 25.3 IRFs. (b) Agreement between Graders 1 and 2 based on area of annotated IRFs in pixels was also good with mean difference of 2091.3 pixels.

Fifteen scans comprising the training dataset were annotated by two separate graders (G1 and G2).  Table 3 shows the number of IRFs annotated by each grader resulting in a total of 9,457 annotated IRFs. Grader 1 annotated a mean ± SD of 302.6 ± 349.1 and Grader 2 annotated a mean ± SD of 327.9 ± 368.1 IRF regions. The agreement of the manual IRF annotation between Graders 1 and 2 was good with a mean difference of 25.3 IRF regions as shown in Figure 5(a) in addition to Pearson's *r* = 0.98 (*P* < 0.0001). Furthermore, there was *κ* = 0.76 between the two graders based on total IRF annotation.

This is expanded upon in Table 4 in which the difference in total annotated IRFs is presented between the two graders. The total difference in annotated scans between the two graders was 629 IRF regions with a mean ± SD of 41.9 ± 45.2 IRF regions.

A challenging aspect of IRF annotation is poor distinction between IRF regions. This may result in one observer annotating one large IRF and another observer annotating multiple smaller IRFs. Thus we analyze the pixel wise area of the annotated IRF regions, presented in Table 5.

Between the two graders, the total annotated IRF area was 3,833,289 pixels, Grader 1 annotated IRFs comprised 1,900,960 pixels, and Grader 2 annotated IRFs comprised 1,932,329 pixels, with an intersecting area of 1,447,480 pixels. As shown in Figure 5(b), agreement between the two graders was again good based on IRF area with a mean difference of 2091.3 pixels, in addition to Pearson's *r* = 0.99 (*P* < 0.0001). Furthermore, there was *κ* = 0.86 between the two graders based on annotated IRF pixel wise area.

Grader reproducibility is further assessed using Hausdorff distance [19] computation between annotated IRF point sets, shown in Table 6. The mean Hausdorff distance ± SD between the two graders was 34.71 ± 30.98 pixels.

## 4. Discussion

The resulting manual IRF annotations obtained in this study must be fit for purpose as a reference standard for both IRF segmentation training and validation. That is to say, not only is it necessary for annotations to be accurate to the position and delineation of the objects in question, but also in the case of the training dataset where annotation was performed by two graders, the annotations must be similar. The first major contribution of this work is a dataset comprised of multidevice SD-OCT scans representative of IRF compositions seen in exudative macular disease. This dataset was annotated by trained graders at the OPTIMA Lab using a predefined annotation criteria based on observed IRF characteristics, described in Section 2.2. A standardized evaluation framework comprised of 4 key measures (Section 2.3) was created to evaluate IRF segmentation algorithms trained using the aforementioned dataset.

Manual delineation of intraretinal cystoid fluid is an extremely time-consuming and difficult task. However, accurately and reproducibly segmented IRFs are necessary as they provide clinically significant information regarding the development, progression, and treatment success of patients with exudative macular disease. As shown in [4], IRFs are an important spatiotemporal feature for longitudinal and cross-patient disease analysis in diseases such as RVO and neovascular AMD. For such purposes, larger datasets are required from which such features are extracted; thus in “big data” situations, there is a need for automated methods of feature extraction (such as IRFs). Furthermore, accurate delineation of features allows the implementation of semisupervised and weakly supervised learning techniques [22] to be applied to “big data.”

Our findings show that, given the criteria of shape/intensity, distinction, continuity, and position describing IRFs, it is possible to annotate these regions reproducibly by two trained graders who are masked to each other. This is exemplified by the high degree of intersection between the two graders with respect to IRF annotation area (>75% intersection pixels) and correlation coefficients 0.98 and 0.99 for IRF region and IRF area, respectively. Furthermore, grader agreement was good exemplified by high *κ*.

The difference in total annotated IRFs between Graders 1 and 2 is shown in Table 4 (calculated from the total annotated IRFs by each grader shown in Table 3) ranging from 1 to over 150 objects. This large range is possibly a result of the subjective nature of human observer annotation despite the presence of guidelines. For example, one grader may judge an object as 1 large IRF, whereas another grader may delineate it as a series of smaller IRFs with a minimal distance between region boundaries. Another possible explanation for IRF region variability is related to the device. Of note is the mean ± SD difference in annotated IRFs by vendor showing that in the case of the Heidelberg Spectralis scans, where the presence of noise is lower due to the averaging of multiple B-scan acquisitions and motion correcting eye tracker is lowest. This value increases for Zeiss Cirrus scans and continues to do so for Topcon 3D 2000 and Nidek RS3000, respectively. This trend correlates with the observed change in image quality in combination with increasing speckle noise (Figure 1), increasing the difficulty for human observers to accurately and reproducibly annotate IRFs.

Thus the number of annotated IRFs is not a representative measure of actual IRF composition and is less suitable for calculating intergrader reproducibility. A more accurate and precise measure is the total object area in pixels annotated by each grader. This can be seen in Table 5 in addition to the total intersection area for each scan, representing the voxels annotated by both graders. As can be seen, in 10 of 15 cases, the difference between graders' total IRF areas was less than 10% of the respective total IRF area for a given scan. In addition, 4 cases were calculated with a difference between grader IRF areas below 5% of the respective total IRF area. This figure rises to 14 out of 15 cases when the threshold is raised to 20% of total annotated IRF area by each grader. Furthermore, examination of the multigrader annotated training dataset Hausdorff distance (Table 6), examining if an annotated voxel from Grader 1 is close to an annotated point from Grader 2, results in a mean ± SD Hausdorff distance of 34.22 ± 30.98 pixels. Again, this is noticeably lower for Spectralis scans (15.74 ± 16.02 pixels) where image quality is better which is to be expected as grader delineation difficulty is lower, compared to Cirrus (27.61 ± 22.13 pixels), Topcon (41.48 ± 14.18 pixels), and Nidek (60.46 ± 58.24 pixels), correlating with their respective levels of noise and poorer image quality. This is the same trend seen in the analysis of total IRF objects annotated from each device. Despite this, the mean Hausdorff distance is still low, indicating a good correlation between graders.

To the best of the authors' knowledge, the dataset presented here is the only publically available dataset comprised of expertly manually annotated intraretinal fluid in SD-OCT scans from multiple vendor devices. The high reproducibility we have shown between grader annotations for each scan in the training dataset is a major advantage of a training dataset annotated by multiple graders as this demonstrates good accuracy and precision. Furthermore, this makes this dataset suitable and fit for use as accurate and reproducible reference standard for the development of retinal IRF segmentation algorithms. In addition, this has also shown that annotation by a single grader examined here is sufficient for use in algorithm testing based on the inclusion criteria describing the IRFs. As such, the testing dataset described in Table 1 has been annotated by a single expert grader per scan and as mentioned previously is intended for testing of developed methods.

## Figures and Tables

**Table 1:** Dataset composition showing total scans of each scanner vendor within each dataset.

Set	Spectralis scans	Cirrus scans	Topcon scans	Nidek scans	Total scans
Training	4	4	4	3	15
Testing	4	4	4	3	15

**Table 2:** Vendor specific small cyst size dimensions in micrometers (width × height).

	Spectralis	Topcon	Cirrus	Nidek
Size *µ*m (width × height)	58.08 × 19.36	39.00 × 13.00	29.33 × 9.775	63.23 × 21.08

**Table 3:** Annotated IRFs by Grader 1 (G1) and Grader 2 (G2) training scans 1 to 4 for each vendor.

Set	Spectralis		Cirrus		Topcon		Nidek		Mean ± SD total IRFs
	G1	G2	G1	G2	G1	G2	G1	G2	
Training 1	128	129	39	46	399	547	299	323	238.8 ± 182.4
Training 2	16	19	69	77	1,170	1,276	258	353	404.8 ± 519.5
Training 3	136	115	995	928	455	409	370	523	491.4 ± 324.3
Training 4	55	47	18	27	132	99	n/a	n/a	63 ± 44.04

Mean ± SD total IRFs	80.63 ± 51.54		274.9 ± 424.6		560.9 ± 437.6		354.3 ± 91.67		

**Table 4:** Difference in number of annotated IRFs between Grader 1 and Grader 2 in the training set scans 1 to 4 for each vendor.

Set	Spectralis	Cirrus	Topcon	Nidek	Mean diff. ± SD (IRFs)
Training 1	1	7	48	24	20 ± 21.06
Training 2	3	8	106	95	53 ± 55.07
Training 3	21	67	46	153	71.75 ± 57.34
Training 4	8	9	33	n/a	16.67 ± 14.15

Mean diff. ± SD (IRFs)	8.25 ± 8.99	22.75 ± 29.51	58.25 ± 32.52	90.67 ± 64.61	

**Table 5:** Total IRF area in pixels annotated by each grader in the training (Trn) set including total number of pixels intersecting (*∩*).

Set	Spectralis (area)		Cirrus (area)		Topcon (area)		Nidek (area)		Mean diff. ± SD (area)
	G1	G2	G1	G2	G1	G2	G1	G2	
Trn. 1	43,986	51,895	24,549	30,017	121,654	128,716	161,714	149,601	8138 ± 2,837
	*∩* = 38,197		*∩* = 18,840		*∩* = 85,268		*∩* = 126,594		

Trn. 2	7,699	8,030	101,264	100,865	400,826	459,439	165,165	157,524	16,746 ± 28,121
	*∩* = 7,114		*∩* = 89,619		*∩* = 291,832		*∩* = 120,507		

Trn. 3	63,879	67,666	386,812	361,372	264,401	257,270	77,549	72,662	10,311 ± 10,181
	*∩* = 44,865		*∩* = 284,912		*∩* = 221,973		*∩* = 49,732		

Trn. 4	7,576	8,361	3,734	4,623	70,152	74,288	n/a	n/a	1,937 ± 1,905
	*∩* = 6,619		*∩* = 2,716		*∩* = 58,692				

Mean diff. ± SD (area)	3,203 ± 3,492		8,049 ± 11,817		19,236 ± 26,289		8,213 ± 3,647		

**Table 6:** Hausdorff distance between grader annotations in pixels.

Set	Spectralis (pixels)	Cirrus (pixels)	Topcon (pixels)	Nidek (pixels)	Mean dist. ± SD (pixels)
Training 1	37.42	18.92	48.51	123.1	56.99 ± 45.75
Training 2	3.162	14.79	52.43	8	19.56 ± 22.40
Training 3	18.28	60.70	44.15	50.25	43.34 ± 18.06
Training 4	4.123	16.03	20.83	n/a	55.12 ± 78.25

Mean dist. ± SD (pixels)	15.74 ± 16.02	27.61 ± 22.13	41.48 ± 14.18	60.46 ± 58.24	
